# Itch Matrixes

**DOI:** 10.3389/fmed.2021.636904

**Published:** 2021-02-24

**Authors:** Peyman Najafi, Laurent Misery, Jean-Luc Carré, Douraied Ben Salem, Olivier Dufor

**Affiliations:** ^1^Univ Brest, LIEN, Brest, France; ^2^Paris-Saclay Institute of Neuroscience, Centre National de la Recherche Scientifique/Université Paris-Saclay, Gif-sur-Yvette, France; ^3^University Hospital of Brest, Department of Dermatology, Brest, France; ^4^Univ Brest, LATIM, INSERM UMR, Brest, France; ^5^University Hospital of Brest, Department of Radiology, Brest, France; ^6^L@bISEN Yncréa Ouest, ISEN, Brest, France

**Keywords:** itch, matrix, pain, brain, imaging

## Introduction

In a recent meta-analysis from our group based on a systematic review we have identified brain regions reported to be responsible for central mechanisms of itch processing (1). We also have discussed the central mechanisms of itch proceeding in the brain more in depth in a review paper (2). The research papers that have studied central mechanism of itch are presented in Table 1 while their results are presented in Table 2. Here in this paper, we are exploring a new idea in which we categorize the itch matrix in the brain into three matrixes that each of them is contributing to a specific aspect of itch perception. This conceptualizes the processing of itch signals into different itch matrices could be useful in order to model different aspects of itch. For example, it is possible, that an overactivity in second matrix cause a higher susceptivity to contagious itch.

**Table 1:** Papers and methods which have been used in order to study central mechanism of itch.

**#**	**References**	**Scanner**	**Neuroimaging analysis**	**Itch induction**	**Itch stimulus**	**Number of subjects**	**Pathology**	**Comments**
1	Hsieh et al. (3)	PET	Subtraction	Intracutaneous injection	Histamine	10	Healthy	
2	Darsow et al. (4)	PET	Subtraction	Skin prick	Histamine	6	Healthy	
3	Darsow et al. (4)	PET	Correlation	Skin prick	Histamine	6	Healthy	
4	Drzezga et al. (5)	PET	Correlation	Skin prick	Histamine	6	Healthy	
5	Mochizuki et al. (6)	PET	Subtraction	Iontophoresis	Histamine	15	Healthy	
6	Walter et al. (7)	fMRI	Correlation	Skin prick	Histamine	6	Healthy	
7	Herde et al. (8)	fMRI	Subtraction	Intracutaneous microdialysis	Histamine	10	Healthy	
8	Leknes et al. (9)	fMRI	Correlation	Skin prick	Histamine	8	Healthy	
9	Leknes et al. (9)	fMRI	Correlation		Allergan	8	Atopic cohort	
10	Mochizuki et al. (10)	fMRI	Correlation	Iontophoresis	Histamine	14	Healthy	
11	Mochizuki et al. (10)	fMRI	Subtraction	Iontophoresis	Histamine	14	Healthy	
12	Valet et al. (11)	fMRI	Subtraction	Skin prick	Histamine	12	Healthy	
13	Valet et al. (11)	fMRI	Subtraction	Skin prick	Histamine	12	Healthy	Temperature modeling
14	Schneider et al. (12)	PET	Subtraction	Iontophoresis	Histamine	6	Healthy	
15	Schneider et al. (12)	PET	Subtraction	Iontophoresis	Histamine	8	Atopic dermatitis	
16	Schneider et al. (12)	PET	Subtraction	Iontophoresis	Histamine	8	Healthy < > AD	
17	Yosipovitch et al. (13)	fMRI	Subtraction	Scratching		13	Healthy	
18	Ishiuji et al. (14)	fMRI	ASL	Iontophoresis	Histamine	8	Atopic dermatitis	
19	Ishiuji et al. (14)	fMRI	ASL	Iontophoresis	Histamine	7	Healthy	
20	Ishiuji et al. (14)	fMRI	ASL	Iontophoresis	Histamine	7	Healthy < > AD	
21	Mochizuki et al. (15)	fMRI	Subtraction	Electrically induced itch		10	Healthy	
22	Mochizuki et al. (15)	MEG	Subtraction	Electrically induced itch		10	Healthy	
23	Vierow et al. (16)	fMRI	Subtraction	Scratching		15	Healthy	
24	Vierow et al. (16)	fMRI	Subtraction	Scratching in presence of itch		15	Healthy	
25	Pfab et al. (17)	fMRI	Subtraction	Skin prick non lesion skin	Histamine	13	Atopic dermatitis	Thermal modulation
26	Pfab et al. (17)	fMRI	Subtraction	Skin prick lesion skin	Histamine	13	Atopic dermatitis	Thermal modulation
27	Bergeret et al. (18)	PET	Subtraction	Iontophoresis	Histamine	28	Healthy	
28	Bergeret et al. (18)	PET	Correlation	Iontophoresis	Histamine	29	Healthy	Itch sensation
29	Holle et al. (19)	fMRI	Subtraction	Audiovisual itch		18	Healthy	
30	Holle et al. (19)	fMRI	Correlation	Audiovisual itch		19	Healthy	
31	Kleyn et al. (20)	fMRI	Subtraction	Skin prick	Histamine	16	Healthy	
32	Kleyn et al. (20)	fMRI	Correlation	Skin prick	Histamine	16	Healthy	
33	Papoiu et al. (21)	fMRI	ASL	Iontophoresis	Histamine	15	Healthy	
34	Papoiu et al. (21)	fMRI	ASL	Spicules rubbing	Cowhage	15	Healthy	
35	Papoiu et al. (21)	fMRI	ASL			15	Healthy	Cowhage < >Histamine
36	Papoiu et al. (21)	fMRI	Subtraction	Audiovisual pain		18	Healthy	
37	Papoiu et al. (21)	fMRI	Subtraction	Itch & Pain		18	Healthy	Itch & Pain
38	Papoiu et al. (22)	fMRI	ASL-correlation	Scratching		14	Healthy	Correlated with the pleasurability
39	Papoiu et al. (22)	fMRI	ASL-correlation	Scratching		14	Healthy	Correlated with itch relief
40	Stumpf et al. (23)	fMRI	Subtraction	Microdialysis	Histamine	33	Healthy	Female>Males
41	Stumpf et al. (23)	fMRI	Subtraction	Microdialysis	Histamine	33	Healthy	Female>Males (with stroop task)
42	Napadow et al. (24)	fMRI	Subtraction	Skin prick	Allergen-induced	14	Atopic dermatitis	Temperature modeling
43	Napadow et al. (24)	fMRI	Subtraction	Skin prick	Allergen-induced	14	Atopic dermatitis	Temperature modeling and acupuncture intervention
44	Desbordes et al. (25)	fMRI	Connectivity	Skin prick	Allergen-induced	14	Atopic dermatitis	Right premotor as seed
45	Desbordes et al. (25)	fMRI	Connectivity	Skin prick	Allergen-induced	14	Atopic dermatitis	Right insula as seed
46	Desbordes et al. (25)	fMRI	Connectivity	Skin prick	Allergen-induced	14	Atopic dermatitis	Right putamen as seed
47	Desbordes et al. (25)	fMRI	Connectivity	Skin prick	Allergen-induced	14	Atopic dermatitis	Left superior parietal lobule as seed
48	Desbordes et al. (25)	fMRI	Connectivity	Skin prick	Allergen-induced	14	Atopic dermatitis	Right anterior mid-cingulate cortex as seed
49	Desbordes et al. (25)	fMRI	Connectivity	Skin prick	Allergen-induced	14	Atopic dermatitis	Right caudate as seed
50	Desbordes et al. (25)	fMRI	Connectivity	Skin prick	Allergen-induced	14	Atopic dermatitis	Right globus pallidus
51	Mochizuki et al. (26)	fMRI	Subtraction	Electrically induced itch		16	Healthy	
52	Mochizuki et al. (26)	fMRI	Subtraction	Electrically induced itch	Passive scratching	16	Healthy	Scratching itch
53	Mochizuki et al. (26)	fMRI	Subtraction	Electrically induced itch	Passive scratching	16	Healthy	Scratching itch> scratching another region
54	Mochizuki et al. (26)	fMRI	Subtraction	Electrically induced itch	Passive scratching	16	Healthy	Deactivation scratching itch region
55	Mochizuki et al. (26)	fMRI	Subtraction	Electrically induced itch	Passive scratching	16	Healthy	Scratching another region
56	Papoiu et al. (27)	fMRI	ASL	Iontophoresis	Histamine	13	End-stage renal disease	
57	Papoiu et al. (27)	fMRI	ASL	Spicules rubbing	Cowhage	13	End-stage renal disease	
58	Kim et al. (28)	fMRI	Subtraction	Audiovisual itch		14	Neurodermatosis	Stress-induced pruritus
59	Kim et al. (28)	fMRI	Subtraction	Audiovisual itch		14	Neurodermatosis	Stress-induced pruritus (after sedating antihistamine treatment)
60	Kim et al. (28)	fMRI	Subtraction	Audiovisual itch		14	Neurodermatosis	Stress-induced pruritus (after non-sedating antihistamine treatment)
61	Mochizuki et al. (29)	fMRI	ASL	Spicules rubbing	Cowhage	10	Healthy	Scratching
62	Mochizuki et al. (29)	fMRI	ASL	Spicules rubbing	Cowhage	10	Chronic itch patients	Scratching
63	Mochizuki et al. (29)	fMRI	ASL	Spicules rubbing	Cowhage	20	Patients>Healthy	Scratching
64	Mochizuki et al. (29)	fMRI	ASL	Spicules rubbing	Cowhage	10	Healthy	Scratching
65	Mochizuki et al. (29)	fMRI	ASL	Spicules rubbing	Cowhage	10	Chronic itch patients	Scratching
66	Mochizuki et al. (29)	fMRI	ASL	Spicules rubbing	Cowhage	20	Patients>Healthy	Scratching
67	Napadow et al. (30)	fMRI		Skin prick	Allergan	14	Atopic dermatitis	Nocebo > open saline
68	Papoiu et al. (31)	fMRI	ASL	Iontophoresis	Histamine	24	Healthy	Areas significantly activated during the suppression of histamine itch by butorphanol
69	Papoiu et al. (31)	fMRI	ASL	Spicules rubbing	Cowhage	25	Healthy	Deactivation areas significantly correlated with the reduction in cowhage itch
70	Vierow et al. (32)	fMRI	Subtraction	Spicules rubbing	Capsaicin	16	Healthy	Placebo
71	Vierow et al. (32)	fMRI	Subtraction	Spicules rubbing	Capsaicin	16	Healthy	Naltrexone
72	Vierow et al. (32)	fMRI	Subtraction	Spicules rubbing	Histamine	16	Healthy	Placebo
73	Vierow et al. (32)	fMRI	Subtraction	Spicules rubbing	Histamine	16	Healthy	Naltrexone
74	Schut et al. (33)	fMRI	ASL-Subtraction	Audiovisual		11	Atopic dermatitis	
75	Schut et al. (33)	fMRI	ASL-correlation	Audiovisual		11	Atopic dermatitis	
76	Stumpf et al. (34)	fMRI	Subtraction	Microdialysis	Histamine	33	Healthy	Itch modulation by distraction (Itch>stroop)
77	van de Sand et al. (35)	fMRI	Subtraction	Skin patch	Histamine	30	Healthy	Nocebo modulation Itch-nocebo > itch only (temperature modulating)
78	van de Sand et al. (35)	fMRI	Connectivity with insula	Skin patch	Histamine	30	Healthy	Nocebo modulation Itch-nocebo > itch only (temperature modulating)
79	Wang et al. (36)	fMRI	Resting state			40+40	Chronic urticaria +Healthy	CSU > HC (amplitude of low frequency fluctuations)
80	Wang et al. (36)	fMRI	Resting state			40+40	Chronic urticaria +Healthy	CSU > HC (functional connectivity with right ventral striatum)
81	Wang et al. (36)	fMRI	Resting state			40+40	Chronic urticaria +Healthy	CSU > HC (functional connectivity with right putamen)
82	Wang et al. (37)	fMRI	Resting state			40+40	Chronic urticaria +Healthy	CSU > HC (regional homogeneity)
83	Wang et al. (37)	fMRI	Resting state			40	Chronic urticaria	After intervention > Before intervention (regional homogeneity)
84	Wang et al. (37)	fMRI	Resting state			40+40	Chronic urticaria +Healthy	CSU > HC (functional connectivity with Cerebellum)
85	Wang et al. (37)	fMRI	Resting state			40	Chronic urticaria	After intervention > Before intervention (functional connectivity with Cerebellum)
86	Wang et al. (37)	fMRI	Resting state			40	Chronic urticaria	After intervention > Before intervention (functional connectivity with SI/MI/SMA)
87	Min et al. (38)	fMRI	Resting state	Skin prick	Histamine	20	Healthy	Acupuncture (itch-baseline)> Non-responder (itch-baseline) (functional connectivity with left Putamen)
88	Min et al. (38)	fMRI	Resting state	Skin prick	Histamine	20	Healthy	Acupuncture (itch-baseline)> Non-responder (itch-baseline) (functional connectivity with right Putamen)
89	Min et al. (38)	fMRI	Resting state	Skin prick	Histamine	20	Healthy	Acupuncture (itch-baseline)> Non-responder (itch-baseline) (functional connectivity with Pallidum)
90	Mochizuki et al. (39)	fMRI	Subtraction	Electrically induced itch		25	Healthy	
91	Mochizuki et al. (39)	fMRI	Connectivity	Electrically induced itch		25	Healthy	

**Table 2:** Results of the all the papers studied the central mechanism of itch.

	**Parts of which matrix**	**1**	**1**				**1/2**	**3**	**2**	**3**	**3**	**3**				**2**	**1**		**2**			**2**									**2**		**2**	
	**Regions**	**Primary somatosensory cortex (BA 1, 2, 3)**	**Somatosensory associated/parietal cortex (BA 5, 7)**	**Primary motor cortex (BA 4)**	**Pre- motor and supplementary motor cortex (BA 6)**	**Cerebellum**	**Insular cortex (BA 13, 16)**	**Posterior cingulate cortex (BA 23,31)**	**Anterior cingulate cortex (BA 24, 32, 33)**	**Prefrontal cortex (BA 9)**	**Frontopolar and orbifrontal area (BA 8, 10, 11, 12)**	**Inferior and dorsolateral prefrontal cortex (BA 44, 45, 46, 47)**	**Temporal gyrus (BA 20, 21, 22, 38)** **+** **fusiform**	**Prietal pole/Wernicke's area (BA 39, 40) Inferior parietal, supramarginal**	**Thalamus**	**Basal ganglia**	**Secondary somatosensory cortex (BA 40, 43) OPC**	**Precuneus (BA 7, 31)**	**Putamen**	**Visual association gyrus (BA 17, 18, 19) ocipital**	**Anterior entorhinal cortex (BA 34)**	**Hippocampus**	**Parahippocampal gyrus**	**Ventral tegmental area cum om Ventral tegmental area**	**Raphé nucleus**	**Red Nucleus**	**PAG**	**Substantia nigra**	**Clastrum**	**Midbrain**	**Amygdala**	**Brain steem**	**Lentiform nucleus**	**Pons**
**#**	**Author**																																	
1	(3)				B	B			C	B																								
2	Darsow et al. (4)	B		C	B				C	C		C	C																					
3	Darsow et al. (4)	B	B	B	C		C		B	C	C	C	C	B			I			C														
4	Drzezga et al., (5)																																	
5	Mochizuki et al. (6)		I		I				C			B		I	C																			
6	Walter et al. (7)					C			C		B		C																					
7	Herde et al. (8)	C	C		B	B	B		B (m)	B	C	B	B	B	B	B																		
8	Leknes et al. (9)						B		B						B	I																		
9	Leknes et al. (9)	C		C	C				B		B			C	B	B																		
10	Mochizuki et al. (10)				I		B	C	C							I																		
11	Mochizuki et al. (10)				I		B		C							I																		
12	Valet et al. (11)	B-		C-	I		C		I-		B-	B		B	B																			
13	Valet et al. (11)			C-	I		B				I-			B																				
14	Schneider et al. (12)	C	C		C			C		I																								
15	Schneider et al. (12)				B	B	I			I	B	C		C	C	I																		
16	Schneider et al. (12)														C	I																		
17	Yosipovitch et al. (13)	B-		B-	B,B-	B	B	B-	I,B-		I,B-	B	B	B			B	B-																
18	Ishiuji et al. (14)		B		B		I	B	B	B	B	B		I		C		B	C	B	C													
19	Ishiuji et al. (14)	C	C	C										C				C																
20	Ishiuji et al. (14)							C										C		C														
21	Mochizuki et al. (15)				C	I	B	I	I			C		B	B		B	C																
22	Mochizuki et al. (15)						B										B	C																
23	Vierow et al. (16)	B		B	B	B	B	B	B	B		B			I		B		B															
24	Vierow et al. (16)	B		B	B	B	B	B	B	B	B	B			B		B		B															
25	Pfab et al. (17)	B-		B-			B-		B-	B		B	B-		B	I																		
26	Pfab et al. (17)	B-	B-		B-		B		B-	B-					B																			
27	Bergeret et al. (18)	I	I			I	C	I					I	B				C		B														
28	Bergeret et al. (18)						C		I																									
29	Holle et al. (19)	L	L		L	B	B					L			B					B														
30	Holle et al. (19)	L			L							L																						
31	Kleyn et al. (20)			I		B			B				C																					
32	Kleyn et al. (20)						C					B	B																					
33	Papoiu et al. (21)																																	
34	Papoiu et al. (21)	C			C	B	C	B	B				C	B	B	B	B	C		B		C	C											
35	Papoiu et al. (21)						B				C	B	C	I			C																	
36	Papoiu et al. (21)				L	B	B			L	L		B		L	B				R														
37	Papoiu et al. (21)				L	B	L			L	L				B	L																		
38	Papoiu et al. (22)							I	B			C	C		B		I	I	I	B		C	C	B	B	B	B	C						
39	Papoiu et al. (22)							C							C	I			I	I														
40	Stumpf et al. (23)				B	B				C	C	B		I		B	C			C, I-														
41	Stumpf et al. (23)				I	C-					C	B	I																					
42	Napadow et al. (24)		I		B		C	C				C				B			B															
43	Napadow et al. (24)	B		B	C		C	I		C						C	C		C	C									C					
44	Desbordes et al. (25)													R				L																
45	Desbordes et al. (25)							L	L				L										L											
46	Desbordes et al. (25)	L			L			B									L (42)																	
47	Desbordes et al. (25)						R				L			L					R															
48	Desbordes et al. (25)						R							R					R															
49	Desbordes et al. (25)											L								L														
50	Desbordes et al. (25)							L				R																						
51	Mochizuki et al. (26)	L		R	R		B	B	R			L			B	B	B	B												B				
52	Mochizuki et al. (26)	B		B	B	B		B	B			B	B	B	B	B	B													B				
53	Mochizuki et al. (26)	B		B	L	L	B		R						L	B		L												L				
54	Mochizuki et al. (26)			B	B	L		L			B							L		B		B												
55	Mochizuki et al. (26)			B	B			R			B							R		B		B												
56	Papoiu et al. (27)	I	I					B	L	B	B	B	B	B				B		I		C	I			B	B	B		I				
57	Papoiu et al. (27)	C		I	B		C	I	B					C	I	C	C	B	C	C		I								C				
58	Kim et al. (28)					R							R	R						L		L												
59	Kim et al. (28)					B			R	L	L		B						L	R			R						L					
60	Kim et al. (28)									B	B		B																					
61	Mochizuki et al. (29)	B	B	B	B	I	B	B	I	B		I			B	B	B										C			C				
62	Mochizuki et al. (29)	C	B	C	B		B	I	I	B		C			B	B	C	I		B										C				
63	Mochizuki et al. (29)			C,I-	B,I-	B-	I-	C				B	C				I-	C																
64	Mochizuki et al. (29)	C	B	C	I	B	B	B	B		B	B			B	I	B	C									B			B				
65	Mochizuki et al. (29)		B	B	B		B	I			I	I	C		B	B	B																	
66	Mochizuki et al. (29)	I	I	C	B	C	C,I-	I			C	I	C		I-	C				I-														
67	Napadow et al. (30)											C		I		C																		
68	Papoiu et al. (31)								I							B																		
69	Papoiu et al. (31)	C				B	I	C					I		I	C						B	B	I		C		I	I					
70	Vierow et al. (32)	B		I-			B	B	B,C-					B	B		B		C			B-									B-			
71	Vierow et al. (32)	B		I-			B	B	B					B	B		B		C			C-												
72	Vierow et al. (32)	B		I-			B	B	B					B	B	C	B		C			C-									B-			
73	Vierow et al. (32)	B		I-			B	B	I					B	B	C	B		C			C-												
74	Schut et al. (33)				B						I					C																		
75	Schut et al. (33)												I				I	I		I														
76	Stumpf et al. (34)				B-		C	C		B-		B-	I-	I	I-			I		B-			C-						I-			I-	B-	
77	van de Sand et al. (35)				C	I					I-					C	I,C-														C			B
78	van de Sand et al. (35)				C-	C	I	I,C-	C				I														I							
79	Wang et al. (36)															R			R															
80	Wang et al. (36)																			R-														
81	Wang et al. (36)				L-																													
82	Wang et al. (37)					B																												
83	Wang et al. (37)	B		B	B																													
84	Wang et al. (37)								B	B	R	R		R		B				R														
85	Wang et al. (37)			R-	R-					R-	R-																							
86	Wang et al. (37)								B-			L-	R-		L-	L-			L-															
87	Min et al. (38)							L															L											
88	Min et al. (38)							L																										
89	Min et al. (38)								C		L	C																						
90	Mochizuki et al. (39)	C	C		C		B	L	B		B	B	B	B		B	B																	

*If laterality applicable: B, Bilateral; C, Contralateral to stimulus; I, Ipsilateral to stimulus. If laterality not applicable: B, Bilateral; L, Left; R, Right*.

*When only the peak locations were reported the sprout022 tool (Dept. of Radiology and Biomedical Imaging, Yale School of Medicine) was used to identify the regions*.

**Figure 1:**
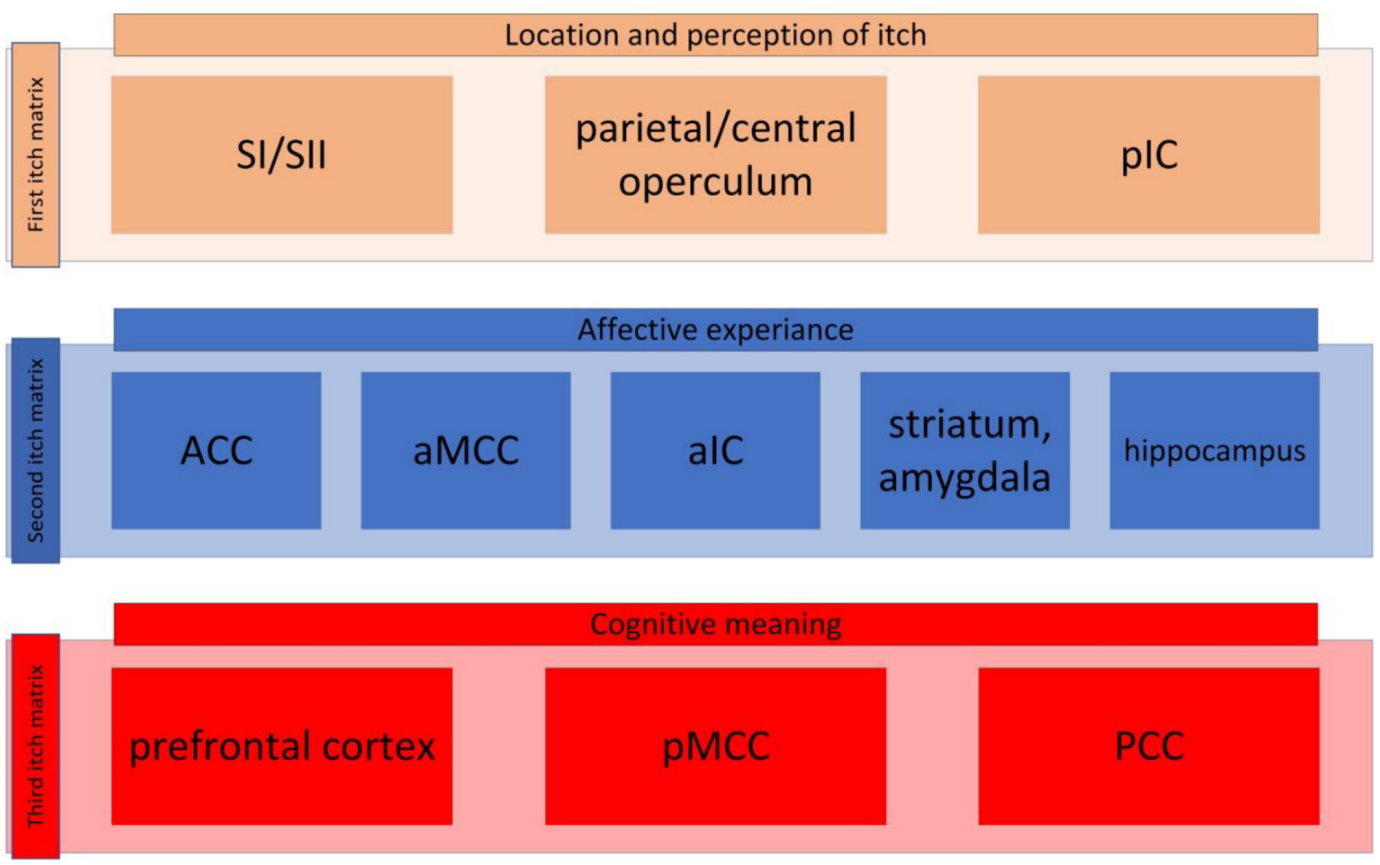
The itch matrix categorized into three itch matrixes. First itch matrix consisted of primary and secondary sensorimotor cortex (SI and SII, respectively), the parietal/central operculum, and the posterior insular cortex (pIC) (here presented in brown, this matrix is also presented in Figure 2A). The second itch matrix consisting of anterior singular cortex (ACC), anterior part of the middle cingulate cortex (aMCC), anterior part of the insular cortex (aIC), amygdala, striatum and hippocampus (here presented in blue, this matrix is also presented in Figure 2B). The third matrix contains prefrontal cortex, posterior part of the middle cingulate cortex (pMCC), and posterior cingulate cortex (PCC) (here presented in red, this matrix is also presented in Figure 2C).

Unlike the visual system pain and itch can evoke multitude of regions in the brain, which we call pain matrix and itch matrix respectively. Recent studies have proposed that the pain matrix can be categorized into three different pain matrixes (40, 41): one contributing to perception and the location of pain; another matrix responsible for the affective aspect of the pain; and a third involving decoding the cognitive aspect of pain. In the same manner, we guardedly propose that the itch processing network can be broken down into three main matrixes although many data are still lacking. These three matrixes have been presented in Figure 1.

## First Itch Matrix

**Figure 2:**
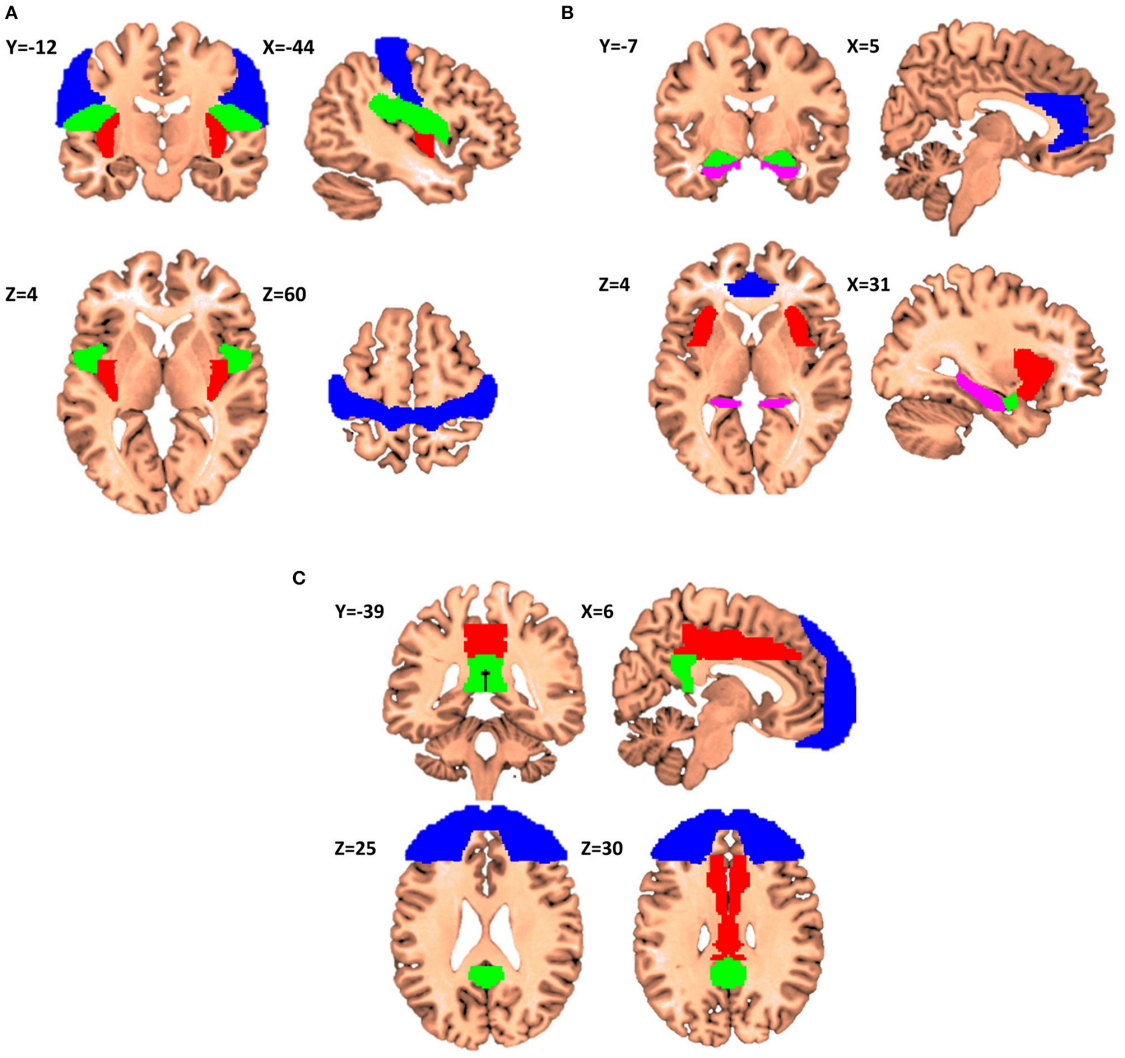
Proposals for itch matrixes (X,Y,Z denotes the location of the corresponding slice in Montreal Neurological Institute (MNI) coordinate system). **(A)** Elements of the first matrix contributing to encoding of the recognition, localization, and intensity of itch. Primary sensorimotor cortex is presented in Blue, parietal operculum in Green, and posterior insular cortex in Red (Regions have been extracted from Automated Anatomical Labeling and Harvard-Oxford atlases). **(B)** The second matrix itch processing matrix consenting of anterior cingulate cortex (Blue), anterior insular cortex (Red), amygdala (Green) and hippocampus (Violet). This matrix is in charge of affective and motivational aspects of itch. **(C)** The third matrix consists of frontal cortex (Blue), middle cingulate cortex (Red), and posterior cingulate cortex (Green), and it is involved in the interpretation of the cognitive meaning of itch.

The first itch matrix includes but is not restricted to the primary sensorimotor cortex, the parietal/central operculum, and the posterior insular cortex (Figure 2A).

Among these three regions the primary sensorimotor cortex is involved in the encoding of the recognition, localization, and intensity of painful stimuli (42). In pain studies, activation in this region bears a linear relationship with pain intensity (43–47). In a positron emission tomography (PET) study by Drzezga et al. (5) the authors reported that SI activity, is positively correlated with itch intensity. Six years after Drzezga, in 2007, Mochizuki et al. added the secondary somatosensory cortex (SII) demonstrating an increase of activity in this region after itch induction with histamine (10). The increase was statistically not different than the proven one observed in the painful condition (pain vs. itch) but did not reach a statistically corrected threshold when comparing itch against no itch.

In another study which includes both AD patients and healthy controls, itch was found to activate the post-central gyrus in the right hemisphere (12). This study together with Drzezga study in 2001 are reported in the meta-analysis on Itch from Lee et al. (48). Out of 56 regions listed in the parietal cortex (31 Left and 25 Right) from 18 studies (Table 1). Brain activity upon itch stimulation in (48), left SI appears to be activated eight times against two only in the right hemisphere. On the contrary, right SII is reported five times against two only in the left hemisphere. The other regions mentioned (*n* = 39) are in both left and right parietal cortices sometime very near to the SI/SII regions (i.e., SMG, SPL, IPL, anterior parietal cortex).

In the meta-analysis from Roberts et al. (49), the authors suggest the possibility of a specificity of these regions for the itching process as they appear to be better activated by itching than by pain. Interestingly, they also group these regions with the central operculum. In a recent meta-analysis of our group (1), SI/SII region was not clearly identified but we discussed this point regarding the diversity of studies we included. Our results on correlations with itch intensity also showed two important clusters in bilateral insular cortices (5068 voxels right 4589 voxels left) that spread to a great extent on the post-central gyri.

The co-activation of the central operculum together with SI/SII cortex is widely reported in itch literature both in healthy subjects and patients. Indeed, central operculum corresponding to the junction of pre- and post-central gyri accompanied with the region located laterally to the posterior convolution of the insula is often confounded with insula itself or even SI. In the regions abbreviated OPC, also named rolandic operculum elsewhere, itch intensity was also correlated with PET signal both in healthy subjects and AD patients (4, 33).

Finally, we propose that the insular cortex, and especially its posterior portion, takes part into this first matrix. As a common point between these regions, their gradual response with itch intensity seems important to highlight. In Leknes et al. bilateral insular and left posterior insular activity (BOLD) is correlated with histamine-induced itch intensity (9). Following Craig (50, 51), Mochizuki et al. postulate that the posterior part of insula plays a different role than its anterior part (52, 53). A distinction that can also find its basis on cytoarchitectural composition of these structures and their connectives with other brain areas (50, 54).

Despite weak evidences in itch literature, other evidences can help to understand the insula role in processing the sensations which are common to itch and pain. Mazzola et al. explain that the two thirds of posterior insula submitted to low electrical stimulation (SEEG) directly translate these stimulations as pain sensations (55). Another study from Frot et al. showed that once pain feeling is reached, the posterior insular cortex activity still correlates with noxious thermal stimulation intensity (47).

In summary, all these regions encode the feeling of itchy sensation and are somewhat translating its intensity level as well as their location following a somatotopic representation. When compared to Xiang et al. study (41), this first matrix includes all already reported regions for pain. However, studies reporting activities in those regions only for itch are rare and some studies need to be carefully interpreted given approximations inherent to main peak reporting. Effectively, secondary peaks of wide clusters or percentage of anatomical regions covered by these clusters are most often not indicated. As an example, the absence of parietal operculum in Roberts et al. study (49) needs to be put in perspective. Indeed, the point that the contrast pain—itch shows an increased activity in the parietal operculum does not mean that this region is silent in itch. Moreover, in the same study, the opposite contrast itch—pain, which reveals an implication of both right supramarginal gyrus and central operculum, could have led us to add more parietal areas to this first matrix.

So far, we have dealt with the membership of each of these brain regions in the matrix separately. However, interesting arguments reside in the fact that new pathological conditions can appear when these regions grouped and malfunction together. Hence, some studies reported that SI/SII together with the insular cortex participate in creating the allodynia phenomenon (56–59). Consecutively, these regions once activated lead to an ignition of the pain network inducing activity in the PAG, the prefrontal cortex, the thalamus, the amygdala, the ACC and many other regions within the pain network. Allodynia has repercussions on the way normal brain areas react to tactile stimuli and authors do not only consider the condition through the scope of pain matrix. Many brain areas are those involved in tactile or thermal sensitivity and this allows more faithful comparison with itch perception. The difficulty with allodynia is that even when it is spontaneous, painful sensation is quickly reached and its intensity then depends on other brain region listed above.

To illustrate this phenomenon, we adduce together both Ducreux et al. study (60) and an article from Geuter et al. (61) about predictive coding. In Ducreux et al. authors demonstrated with noxious and non-noxious cold stimulation (4° and 22°C) that while non-noxious cold in control subjects activates SII and the insular cortex (mostly its anterior part), the same non-noxious stimulation did activates SII and mid-posterior insula in allodynic patients together with other regions of the pain network (60). In Geuter et al. work, the authors used the predictive coding theory of brain functioning to demonstrate a difference within the anterior and the posterior part of the insula. While the anterior part would be dedicated to pain feelings as a prediction error on perceived sensations, the posterior part only responds to pain intensity with no comparisons to any predicted sensation (61). We propose that in Ducreux et al. even if the feeling is non-noxious in control subjects, it remains unpredictable and then activates the anterior part of the insula. However, allodynic patients are prepared to feel painful stimulation and then, the anterior part shut down as painful sensation are correctly predicted. Meanwhile, the posterior part of the insula starts to encode its intensity like it was demonstrated by Frot et al. (47) in implanted subjects when stimulation becomes noxious.

## Second Itch Matrix

The second itch matrix could consist of the ACC, aMCC, aIC, amygdala, striatum and hippocampus (Figure 2B). This network could encode the affective and motivational aspects of itch. Significant activation in the ACC, especially dorsal, extending to the anterior part of the middle cingulate cortex (aMCC), has been linked to the reward network and the positive or negative emotional response (40). Noteworthy, Vogt has reported that the aMCC reflects emotional awareness and fear leading to the questioning of the enrolment of the aMCC to the ACC gross function (62, 63). Considering the anterior insula, it is reported to be involved in the awareness of emotions and subjective feelings (50) as well as errors of predictions like mentioned above. Another literature about lesions in the aIC would cause deficits in emotional awareness (e.g., alexithymia) (64). Several studies have reported that activity in the aIC is significantly correlated with the unpleasantness of itch (8–10, 18, 21). For the hippocampus, it has been also shown that this structure is fully integrated in the itch network (13, 21, 22). For example, only active scratching can relief activity in ipsi-hippocampal structure (53). The role of hippocampus together with amygdala, dACC and insular cortex are well-documented in Sanders and Akiyama (65). The authors noticed and argued that “amygdala and hippocampus activation appears to go hand-in-hand in most studies of itch, suggesting that the memory of previous itch experiences may be a significant factor in itch-related anxiety.” Stratum possibly involved with motivation aspects of itch and/or the carving for scratching.

According to original paradigms, two other studies have reported diminished activation of these regions in tasks that change the nature of pain perception with context variations (66) or with analgesia induced by meditation (67). While the first of these shows a diminished activation in dorsal ACC and insula as the subjects switch their perception from unpleasant to pleasant (or less unpleasant) revealing the link between emotional and motivational function. The second demonstrate that experienced Zen meditators can reduce activity of their prefrontal medial cortex, amygdala and hippocampus regions at the expense of an increased activity in dorsal ACC or insula which still belong to this second matrix but are more related to mindfulness. These articles suggest that making things more conscious by bringing activities closer to the awareness matrix (with insula as a common region) putatively lead to less harmful psychological consequences. This second matrix is more robust than the first one. Many arguments in the itch literature exist and converge about its functional role.

## Third Itch Matrix

The third itch matrix would include parts of the prefrontal cortex, pMCC, and PCC (Figure 2C). This network should be involved in the subjective perception of itch. The cognitive state of the mind can affect the itch sensation e.g., emotions, obsessions, religious beliefs, disgusts, expectations, and past experiences. This pattern of activation is also present in the distraction from itch caused by the Stroop task (e.g., in the DLPFC) (14, 30, 34). The third matrix receives and integrates information from the foregoing two and triggers behavioral response.

## Conclusion

Knowledge of itch processing in the brain is growing thanks to brain imaging (2, 68). A better understanding of interactions between itch matrixes would allow a better understanding of pruritus in different cutaneous or extra-cutaneous etiologies (69).

## Author Contributions

LM, J-LC, DB, and OD contributed to conception and design of the study. PN organized the database and wrote the first draft of the manuscript. OD wrote sections of the manuscript. All authors contributed to manuscript revision, read, and approved the submitted version.

## Conflict of Interest

The authors declare that the research was conducted in the absence of any commercial or financial relationships that could be construed as a potential conflict of interest.

## Abbreviations

ACC: Anterior Cingulate Cortex
AD: Atopic Dermatitis
aIC: Anterior part of the Insular Cortex
aMCC: Anterior part of the Middle Cingulate Cortex
BOLD: Blood Oxygenation Level Dependent
dACC: Dorsal part of the Anterior Cingulate Cortex
DLPFC: Dorso-Lateral Pre-Frontal Cortex
IPL: Inferior Parietal Lobule
OPC: Operculum Central
PAG: Peri-Aqueductal Gray matter
PCC: Posterior Cingulate Cortex
PET: Positron Emission Tomography
pIC: Posterior Insular Cortex
pMCC: Posterior part of the Middle Cingulate Cortex
SI: Primary Somatosensory cortex
SII: Secondary Somatosensory cortex
SEEG: Stereo-Electro-Encephalo-Graphy
SMG: Supra-Marginal Gyrus
SPL: Superior Parietal Lobule.
